# Autofluorescence of NADH is a new biomarker for sorting and characterizing cancer stem cells in human glioma

**DOI:** 10.1186/s13287-019-1467-7

**Published:** 2019-11-20

**Authors:** Ye Yuan, Zexuan Yan, Jingya Miao, Ruili Cai, Mengsi Zhang, Yanxia Wang, Lihong Wang, Weiqi Dang, Di Wang, Dongfang Xiang, Yan Wang, Peng Zhang, Youhong Cui, Xiuwu Bian, Qinghua Ma

**Affiliations:** Institute of Pathology and Southwest Cancer Center, Key Laboratory of the Ministry of Education, Southwest Hospital, Third Military Medical University (Army Medical University), Chongqing, 400038 China

**Keywords:** Glioma stem cells, Autofluorescence, NADH, FACS, Biomarker

## Abstract

**Background:**

The existing cell surface markers used for sorting glioma stem cells (GSCs) have obvious limitations, such as vulnerability to the enzymatic digestion and time-consuming labeling procedure. Reduced nicotinamide adenine dinucleotide (NADH) as a cellular metabolite with property of autofluorescence has the potential to be used as a new biomarker for sorting GSCs.

**Methods:**

A method for sorting GSCs was established according to the properties of the autofluorescence of NADH. Then, the NADH^high^ and NADH^low^ subpopulations were sorted. The stem-like properties of the subpopulations were evaluated by qRT-PCR, western blot analyses, limiting dilution assay, cell viability assay, bioluminescence imaging, and immunofluorescence analysis in vitro and in vivo. The relationship between CD133^+^/CD15^+^ cells and NADH^high^ subpopulation was also assessed.

**Results:**

NADH^high^ cells expressed higher stem-related genes, formed more tumor spheres, and harbored stronger pluripotency in vitro and higher tumorigenicity in vivo, compared to NADH^low^ subpopulation. NADH^high^ glioma cells had the similar stemness with CD133^+^ or CD15^+^ GSCs, but the three subpopulations less overlaid each other. Also, NADH^high^ glioma cells were more invasive and more resistant to chemotherapeutic drug temozolomide (TMZ) than NADH^low^ cells. In addition, the autofluorescence of NADH might be an appropriate marker to sort cancer stem cells (CSCs) in other cancer types, such as breast and colon cancer.

**Conclusion:**

Our findings demonstrate that intracellular autofluorescence of NADH is a non-labeling, sensitive maker for isolating GSCs, even for other CSCs.

## Background

Glioma stem cells (GSCs) are believed to be responsible for tumor initiation, progression, chemo- and radioresistance, and recurrence of gliomas [1–4]. The identification and isolation of GSCs are crucial for a better understanding of their properties and developing GSC-targeting therapies. GSCs are usually identified and isolated from primary tumors or glioma cell lines by fluorescence-activated cell sorting (FACS) based on the cell surface makers, such as CD133 and CD15. Early studies reported that only 100 CD133-positive cells of glioma could produce a phenocopy of parent tumor in NOD-SCID mice, whereas 10^5^ CD133-negative cells could not [1, 5]. CD15 has also been considered as another reliable surface marker for isolating GSCs [6]. However, recent studies indicated that CD133- or CD15-negative glioma cells also possessed some GSC characteristics [6–8]. It is unclear whether partial CD133/CD15-negative cells have the properties of CSCs per se or partial CD133/CD15-negative GSCs are derived from CD133/CD15-positive subpopulation missing the markers by enzymatic digestion [9, 10]. In addition, the antibodies of CD133/CD15 are expensive and the labeling process is time-consuming. Therefore, it is necessary to find alternative strategies, which are more specific, simple, and economic for the isolation of GSCs.

Energy metabolism is involved in the self-renewal, reprogramming, and differentiation of regular stem cells and cancer stem cells (CSCs) [11, 12]. Reduced nicotinamide adenine dinucleotide (NADH) is a key carrier of electrons in cellular energy metabolism. It possesses a property of autofluorescence with an excitation wavelength at 340 ± 30 nm and an emission wavelength within the 460 ± 50 nm range [13, 14], and has been used as an important intracellular autofluorescence component to non-invasively monitor and analyze metabolic activity of living cells and tissues [15, 16]. Recently, NADH fluorescence intensity and fluorescence lifetime of bound and free NADH have been used to distinguish stem cells from their differentiated progeny [17–19]. Besides, NADH has been used to screen or monitor GSC metabolic state by using fluorescence lifetime microscopy (FLIM) [20]. However, the usability of NADH autofluorescence in the isolation and purification of GSCs by FACS has not been evaluated.

In the present study, we applied the autofluorescence of NADH as a non-labeling marker to isolate GSCs by FACS. Compared to NADH^low^ subpopulation, NADH^high^ subpopulation exhibited higher stem-like properties, including abilities of self-renewal, multilineage differentiation, and tumorigenesis, as well as higher invasive ability and resistance to chemotherapeutic temozolomide (TMZ). Besides, NADH^high^ as a biomarker could be used to isolate breast and colon CSCs. Therefore, NADH is a suitable biomarker for the isolation of GSCs or other CSCs.

## Materials and methods

### Human glioma specimens and the preparation of single cell suspension

A total of 13 fresh surgical glioma specimens were collected from patients enrolled in the Southwest Hospital, Third Military Medical University, Chongqing, China, after signing an informed consent from patients or their guardian. All patients had not received chemoradiotherapy before surgery. The histopathological grading was in accordance with the World Health Organization (WHO) classification (2016). The clinicopathologic information of these patients is summarized in Additional file 1: Table S1. This study was approved by the Ethics Committee of Southwest Hospital.

To prepare the single cell suspension, fresh surgical glioma tissues were collected and cut into small pieces immediately, and then, glioma cells were isolated using the Papain Dissociation System (Worthington Biochemical, Lakewood, NJ, USA) as previously reported [21, 22] and suspended in PBS at 1–5 × 10^6^ cells/mL.

### Cell lines and culture

Glioma cell lines (T98G, LN229), breast cancer cell line (MDA-MB-231), and colon cell line (HT-29) were purchased from ATCC (VA, USA). Primary glioma cells GBM1 and GBM2 were respectively isolated from two human glioma surgical specimens in our laboratory [23, 24]. All the cell lines were maintained in DMEM (HyClone, USA) supplemented with 10% fetal bovine serum (FBS) (HyClone, USA). The medium for tumorsphere culture was composed of F12 medium containing 20 μL/mL B27 supplement (Gibco, USA), 20 ng/mL basic fibroblast growth factor (bFGF), and 20 ng/mL epidermal growth factor (EGF) (both from PeproTech, USA) without serum. All the cells were cultured at 37 °C in 5% CO_2_ and 100% humidity.

### FACS analysis and cell sorting

The cultured glioma cells were digested by trypsin or accutase and resuspended with PBS. The fresh glioma specimens were transferred to laboratory on ice in half hour after surgery, then washed and enzymatically dissociated into single cells and resuspended in PBS. The staining procedures for CD133 and CD15 markers were performed as previously described [6, 8]. The labeling antibodies were anti-CD133-APC antibody (Clone REA816; Miltenyi Biotec, Germany) and anti-CD15-FITC antibody (Biolegend, USA) with REA Control (S)-APC (Miltenyi Biotec, Germany) and FITC Mouse IgM (Biolegend, USA) as controls, respectively.

The FACS analysis and cell sorting were performed on BD FACS Aria II cytometer (USA) or Beckman moflo XDP (USA). For analyzing and sorting with NADH autofluorescence intensity as a marker, an excitation wavelength of 375 nm or 355 nm and an emission wavelength of 450/50BP filter were used. For analyzing and sorting with CD133 and CD15 as markers, labeled cells were analyzed and sorted with corresponding excitation and emission wavelengths of the fluorochrome. All data were analyzed with BD FACSDiva software version 8 or Beckman moflo XDP submmit 5.2.

### Limiting dilution assay

Limiting dilution assay was performed as previously described [24]. Briefly, serial twofold dilutions (from 40 to 0 cells) of different glioma, breast cancer, and colon cells were seeded into ultra-low adhesion 96-well plates (10 wells per dilution) (Costar, USA) and cultured in tumorsphere medium. After incubation for 2 weeks, wells without spheres (log2, *Y*-axis) were counted and plotted against the number of cells plated per well (*X*-axis) to calculate the sphere formation efficiency.

### RNA preparation and qRT-PCR

Total RNAs from sorted cells by FACS were extracted with RNA extracting Kit (Fastagen, China) according to the manufacturer’s instructions. One microgram of total RNA was reverse transcribed with the Reverse Transcription Kit (Takara, Dalian, China). Quantitative real-time PCR was carried out using the SYBR PrimeScript PCR kit II (TaKaRa, Japan). The level of β-tubulin mRNA was used as the internal control. The primers used in this study are listed in Additional file 1: Table S2.

### Cell viability assay and IC50 evaluation

Different subpopulations of GBM1 and LN229 cells were seeded in 96-well plates at 2 × 10^3^ cells/well and treated with TMZ at the indicated concentrations for 48 h. The viability of glioma cells was measured by using a Cell Counting Kit-8 (Beyotime, China) according to the manufacturer’s instructions. The OD values at 450 nm were recorded by fluoroanalyzer (Floskan Ascent, USA).

### Immunofluorescence analysis

For induction of differentiation, NADH^high^ cells were cultured in DMEM with 10% FBS for 7 days. The NADH^high^ cells cultured in same conditions within 6 h were used as controls. Both differentiated and control cells were fixed in 4% paraformaldehyde for 30 min, washed three times with PBS at room temperature, and incubated with blocking buffer containing 10% normal goat serum and 0.3% Triton. The samples were incubated with primary antibodies anti-Sox2 (#3579, 1:400, CST), anti-Nestin (#33475, 1:400, CST), and anti-GFAP (#12389, 1:400, CST) overnight at 4 °C. Hoechst 33342 was used to counterstain the cell nuclei. After washing with PBS, the samples were mounted with Immuno-Mount™ (Thermo Scientific, USA) and then examined on a LEICA TCS-SP5 confocal microscope (× 63 objective).

### Xenograft in NOD-SCID mice and bioluminescence imaging

The animal study was performed in accordance with the protocol approved by the Institutional Animal Care and Use Committee of Southwest Hospital, Third Military Medical University (TMMU). NOD/SCID female mice (5 weeks old) were purchased from the Laboratory Animal Center of TMMU. Different treated GBM cells were washed and resuspended in PBS and mixed with Matrigel (1:1, BD Biosciences), then subcutaneously injected into NOD/SCID mice at 4 × 10^3^, 4 × 10^4^, and 4 × 10^5^ cells (100 μL/site) with the left flank as the test group and right flank as the control group. Tumor growth was monitored by bioluminescence imaging using In Vivo Imaging System (IVIS) Spectrum (Perkin Elmer, USA) and Living Image Software for IVIS (Perkin Elmer). At the end of 6 weeks after the injection, the mice were killed. Xenograft tumors were removed and weighted.

### Western blotting

Western blotting was performed as previously described [25]. The primary antibodies used in western blot were anti-Sox2 (#3579, 1:1000, CST), anti-CD133 (#64326, 1:1000, CST), anti-Nanog (#8822, 1:1000, CST, USA), and anti-β-tubulin (#2128, 1:10000, CST).

### Transwell invasion analysis

Glioma cells were seeded into the upper chambers (Millipore, 8.0 μm, 24 well) that were coated with 15 μL/well of Matrigel in advance (Corning, USA) at the density of 3 × 10^4^ cells/well in 200 μL of serum-free DMEM, and then, the upper chambers were placed in a 24-well plate added with 600 μL/well DMEM supplemented with 10% FBS. After incubation for 24 h, the cells were fixed with 4% paraformaldehyde followed by crystal violet staining. Non-invading cells were removed with a cotton swab, and the images of stained cells were collected by microscope (Olympus, Japan).

### Statistical analysis

All experiments were performed at least three times. Statistical analysis was performed by using SPSS statistical software (SPSS16.0, Chicago, CA, USA) and GraphPad Prism 6 software (GraphPad, La Jolla, CA, USA). The unpaired two-group comparison and multiple comparisons were made with Student’s *t* test or one-way ANOVA, respectively. Data were presented as the mean ± SD. Statistical significance was set at *p < 0.05, **p < 0.01, and ***p < 0.001.

## Results

### NADH^high^ and NADH^low^ subpopulations can be sorted from glioma cells by FACS in vitro

**Fig. 1:**
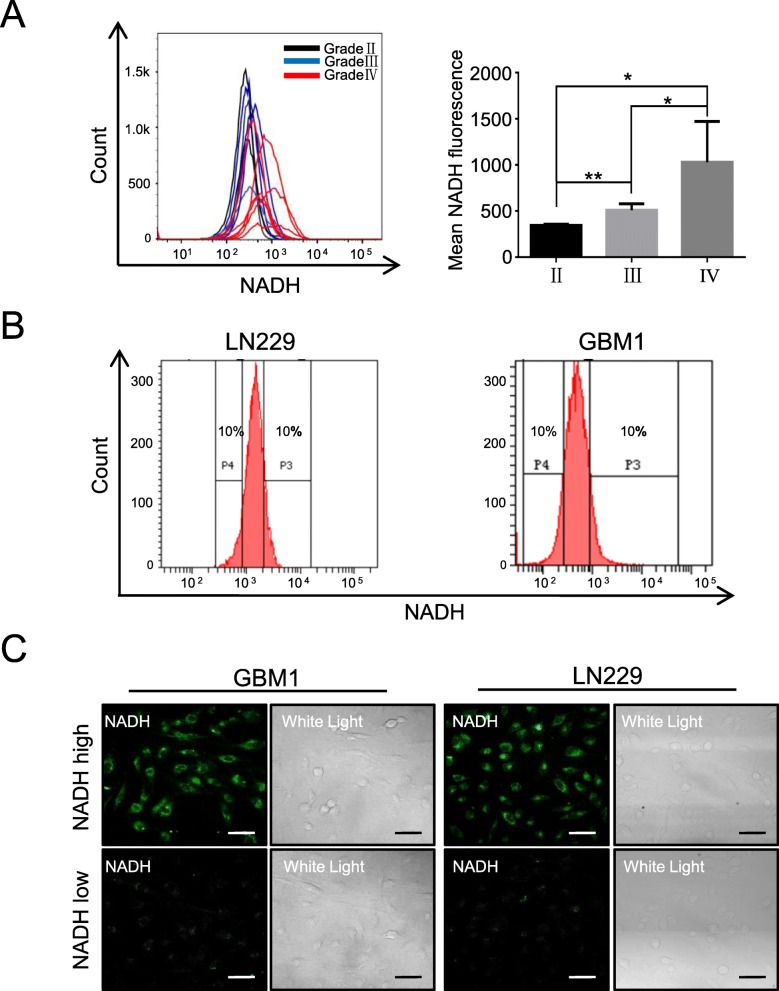
NADH^high^ and NADH^low^ glioma cell subpopulations can be sorted according to their intensity of NADH autofluorescence. **a** The intensity of NADH autofluorescence increased with WHO grades in primary glioma cells. Also, the intensity of NADH autofluorescence in patients within same grade II (*n* = 4) or III (*n* = 3) was similar, but major difference in grade IV (*n* = 6) patients was observed. **b** Glioma cells with the highest top 10% and lowest bottom 10% intensity of NADH autofluorescence were defined as NADH^high^ and NADH^low^, respectively. **c** The intensity of NADH autofluorescence in sorted NADH^high^ and NADH^low^ glioma cells was verified by confocal microscopy. All data are presented as the means ± SD. *p < 0.05, **p < 0.01 (*n* = 3 independent experiments)

By using flow cytometry, we firstly examined the autofluorescence intensity of NADH in 13 fresh glioma tissues, including 4 WHO grade II, 3 grade III, and 6 grade IV. The autofluorescence intensity of NADH was increased with WHO grades (grade IV > grade III > grade II); in low-grade gliomas (grades II and III), the autofluorescence intensity of NADH was similar between the samples, but large difference between samples was observed in grade IV (Fig. 1a, Additional file 1: Figure S1). According to previous reports [26, 27], we defined the highest top 10% intensity as high autofluorescence of NADH (NADH^high^) and defined the lowest bottom 10% intensity as low autofluorescence of NADH (NADH^low^). Accordingly, we sorted the subpopulations with top 10% and bottom 10% intensity of NADH autofluorescence from GBM1 and LN229 cells (Fig. 1b). To confirm the autofluorescence intensity of NADH in both NADH^high^ and NADH^low^ subpopulations, we examined the intensity of NADH autofluorescence with confocal analysis. The cells with top 10% intensity of NADH showed strong autofluorescence intensity, while the cells with bottom 10% intensity of NADH had weak fluorescence signal (Fig. 1c). These results indicate that NADH^high^ and NADH^low^ subsets existed in glioma cells and could be promptly isolated by FACS.

### NADH^high^ glioma cells exhibit GSC traits in vitro

**Fig. 2:**
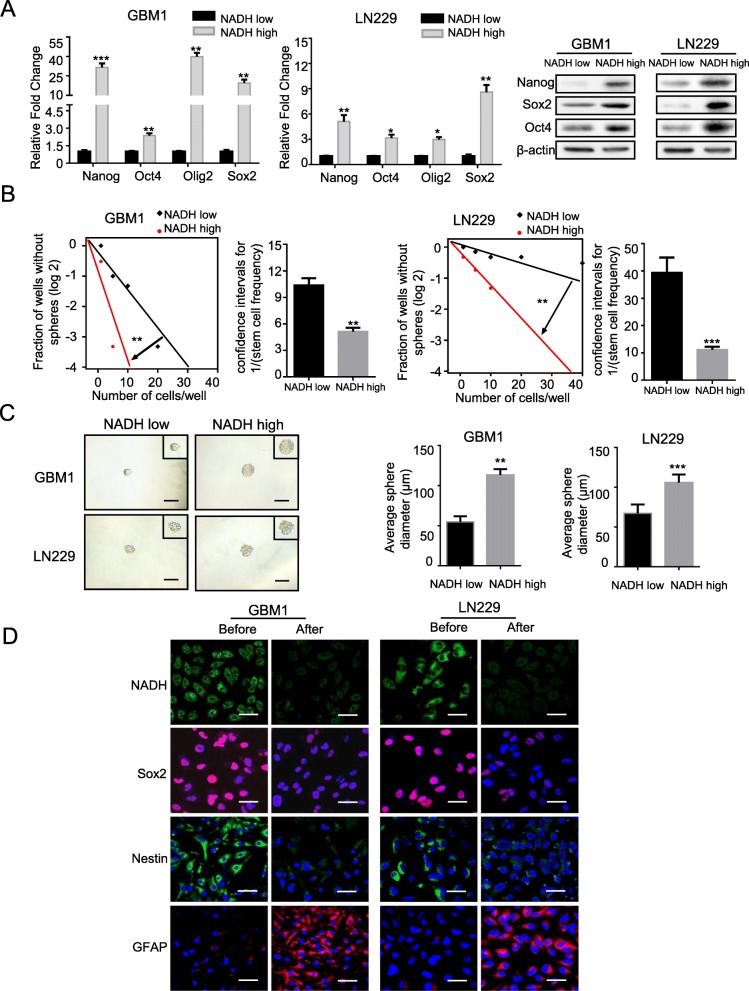
NADH^high^ glioma cells exhibit characteristics of GSCs in vitro. **a** qRT-RCR and western blotting analyses showed higher expression levels of stem-related genes in NADH^high^ cells than in NADH^low^ cells in GBM1 and LN229 cell lines. **b**, **c** Limiting dilution assays showed higher tumorsphere formation rates and longer average diameter of tumorsphere in NADH^high^ cells compared to NADH^low^ cells in GBM1 and LN229 cell lines. **d** Cultured in medium supplemented with 10% FBS for 7 days, NADH^high^ cells markedly reduced the autofluorescence of NADH and the expression of neural stem/progenitor markers Sox2 and Nestin, but re-expressed high astroglial marker GFAP. Scale bar = 50 μm. All data are presented as the means ± SD. *p < 0.05, **p < 0.01, ***p < 0.001 (*n* = 3 independent experiments)

To evaluate the stem-related properties of NADH^high^ and NADH^low^ glioma cells in vitro, we first compared the expression of stem-related genes in both subpopulations. Compared to NADH^low^ subpopulation, NADH^high^ glioma cells highly expressed stem-related genes Nanog, Oct-4, Oligo2, and Sox2 at both mRNA and protein levels in GBM1 and LN229 cells (Fig. 2a). Then, the tumorsphere formation of NADH^high^ and NADH^low^ cells was measured through a limiting dilution analysis. In comparison with NADH^low^ glioma cells, NADH^high^ cells showed higher rate of tumorsphere formation in both GBM1 and LN229 cells (*p* < 0.01 for both) (Fig. 2b). Moreover, the average diameter of the tumorspheres derived from NADH^high^ cells was about twice as much as that derived from NADH^low^ cells in GBM1 and LN229 cells (*p* < 0.01 for both) (Fig. 2c).

Previous studies showed that GSCs harbored multipotency to differentiate into neurons, astrocytes, and oligodendrocytes, and stem cell markers disappeared with the differentiation [28, 29]. Hence, we evaluated whether the NADH^high^ subpopulation had multiple differentiation potential by a differentiation assay. As expected, the differentiated NADH^high^ cells almost lost not only autofluorescence of NADH but also neural stem/progenitor markers Sox2 and Nestin and re-expressed astroglial marker GFAP (Fig. 2d). Thus, these data strongly indicate that NADH^high^ glioma cells have the characteristics of GSCs in vitro.

### NADH^high^ glioma cells show high tumorigenicity in vivo

**Fig. 3:**
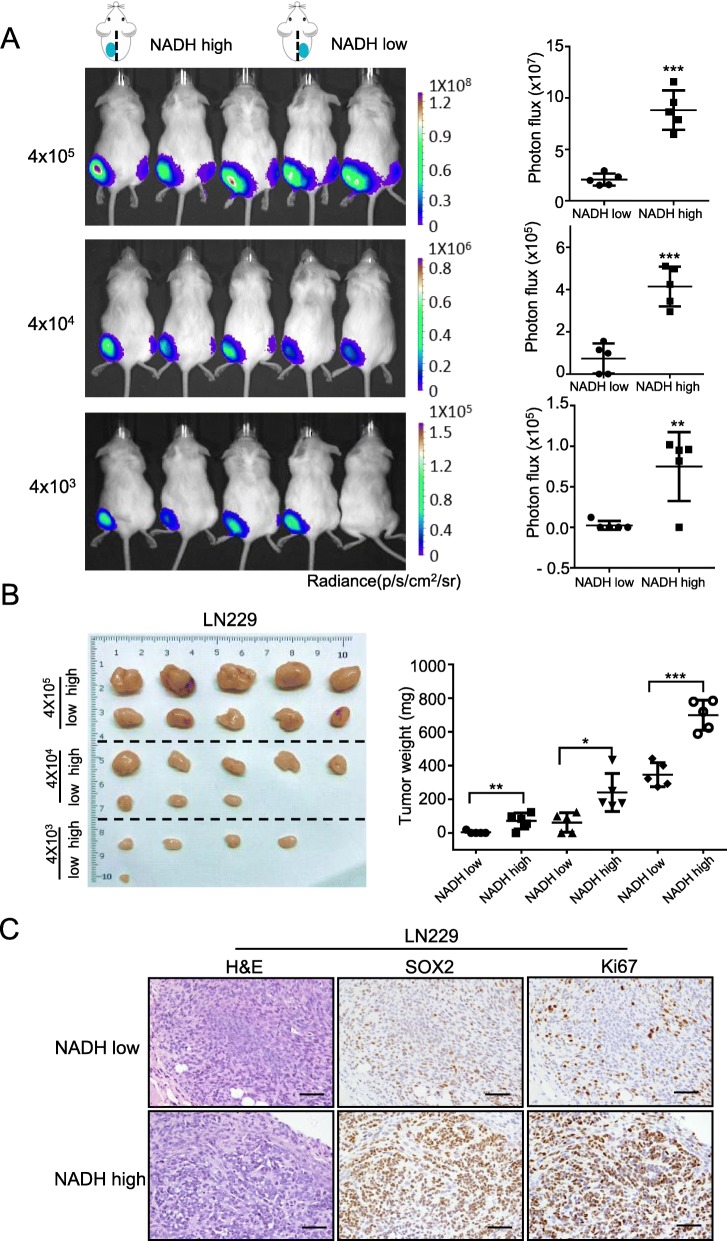
NADH^high^ glioma cells exhibit characteristics of cancer stem cells in vivo. **a** Bioluminescent images and quantification showed that the total flux of the tumors derived from NADH^high^ LN229 cells (left flank) was extremely higher than that of the tumors derived from NADH^low^ LN229 cells (right flank) at 28 days after subcutaneous implantation in NOD/SCID mice. Also, the total photon flux of the tumors was increased with implanted cell number. Signal intensity is represented as p/s/cm^2^/sr. **b** The images of xenograft tumors showed that NADH^high^ LN229 cells had higher rate of tumor formation than NADH^low^ cells (left panel). Weight statistical diagram showed that the weight of NADH^high^ LN229 cell-derived tumors was heavier than that of NADH^low^ LN229 cell-derived tumors (right panel). **c** H&E staining confirmed the glioma origin of the xenograft tumors, and IHC staining showed that NADH^high^ LN229 cell-derived tumors expressed higher expression of Sox2 and Ki67 than NADH^low^ cells. Scale bar = 50 μm. All data are presented as the means ± SD. *p < 0.05, **p < 0.01, ***p < 0.001 (*n* = 3 independent experiments)

The stem-related properties of NADH^high^ and NADH^low^ glioma cells in vivo were further evaluated by xenograft experiment in NOD-SCID mice. Bioluminescent analyses showed that the tumor size derived from NADH^high^ subpopulation was significantly larger than that derived from NADH^low^ subpopulation in LN229 cells at 28 days after implantation (Fig. 3a). As shown in Fig. 3b and Additional file 1: Table S3, the tumor incidence rate of NADH^high^ cells was higher than that of NADH^low^ cells. The weight of tumors derived from NADH^high^ cells was heavier than that derived from NADH^low^ cells (Fig. 3b). H&E staining confirmed the glioma origin of tumors, and IHC showed that the tumors derived from NADH^high^ cells exhibited higher Ki-67 and Sox2 expression than those derived from NADH^low^ cells (Fig. 3c). These results indicate that NADH^high^ glioma cells have high tumorigenicity in vivo.

### NADH^high^ glioma subpopulation possesses similar stem-like properties with CD133^+^ or CD15^+^ cells, but only partially overlaps with them

Since CD133 and CD15 are usually used as makers to enrich GSCs by FACS, the relationship between NADH^high^, CD133^+^, and CD15^+^ subpopulations was assessed. We first measured the proportions of CD133^+^ and CD15^+^cells in GBM1, GBM2, T98G, and LN229 cells and found that the percentages were 0.73 ± 0.04%, 0.47 ± 0.04%, 1.37 ± 0.22%, and 0.53 ± 0.04%, and 0.50 ± 0.07%, 0.1%, 4.77 ± 0.24%, and 0.13 ± 0.09%, respectively (Additional file 1: Figure S2 and Figure S3, Table 1), which was consistent with previous reports [30–32]. We then compared the proportions of CD133^+^ and CD15^+^cells in NADH^high^ and NADH^low^ subpopulations in those glioma cell lines. The percentages of CD133^+^ cells were elevated about two times in NADH^high^ subpopulation, but no obvious change in NADH^low^ subpopulations of those cell lines (Additional file 1: Figure S2, Additional file 1: Table S1). The proportion changes of CD15^+^ cells in NADH^high^ and NADH^low^ subpopulations were similar to those of CD133^+^ cells (Additional file 1: Figure S3, Additional file 1: Table S1). The proportion of NADH^high^ cells in CD133^+^ and CD15^+^ subpopulations was also analyzed. The percentages of NADH^high^ cells ranged from 20.1 to 63.7% in CD133^+^ subpopulation and from 10 to 86.2% in CD15^+^ subpopulation in the four cell lines (Additional file 1: Figure S4). These results suggest that NADH^high^ subpopulation is only partially overlapped with CD133^+^ or CD15^+^ subpopulation.

**Table 1:** The percentage of CD133^+^ and CD15^+^ cells in NADH^high/low^ subpopulations of glioma cell lines

Cell lines	Percentage of CD133^+^ cells			Percentage of CD15^+^ cells		
	Total	NADH^high^	NADH^low^	Total	NADH^high^	NADH^low^
GBM1	0.73 ± 0.04	2.10 ± 0.13	0.60 ± 0.13	0.50 ± 0.07	2.47 ± 0.44	0.43 ± 0.09
GBM2	0.47 ± 0.04	2.63 ± 0.24	0.43 ± 0.11	0.1	0.40 ± 0.13	0
T98G	1.37 ± 0.22	2.27 ± 0.22	0.63 ± 0.31	4.77 ± 0.24	6.40 ± 0.07	6.23 ± 0.51
LN229	0.53 ± 0.04	1.83 ± 0.18	0.80 ± 0.13	0.13 ± 0.04	0.67 ± 0.04	0.27 ± 0.04

**Fig. 4:**
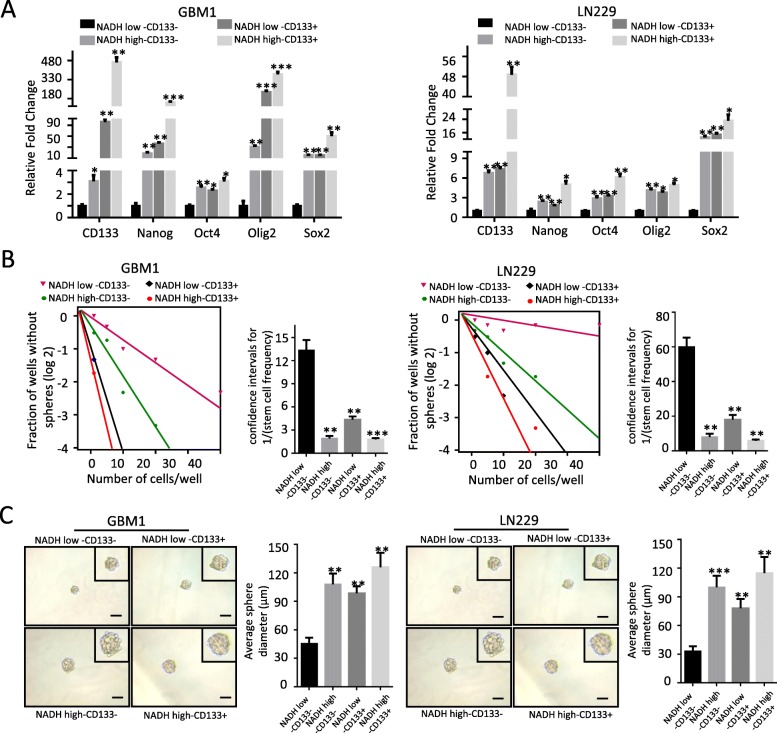
Both NADH^high^ and CD133^+^ glioma cells possess the properties of GSCs, but are independent each other. **a** qRT-PCR analysis showing upregulated stemness-related transcription factor genes Nanog, Oct4, Oligo2, and Sox2 in NADH^high^ CD133^+^, NADH^high^ CD133^−^, and NADH^low^ CD133^+^ subpopulations, compared to NADH^low^ CD133^−^ in GBM1 and LN229. **b**, **c** Limiting dilution assay showed increased sphere formation rate and sphere average diameter of NADH^high^/CD133^+^, NADH^high^/CD133^−^, and NADH^low^/CD133^+^ subpopulations, compared to NADH^low^ CD133^−^cells in GBM1 and LN229 cell lines. All data are presented as the means ± SD. *p < 0.05, **p < 0.01, ***p < 0.001 (*n* = 3 independent experiments)

To further illustrate the relationship among NADH^high^, CD133^+^, and CD15^+^ subpopulations, we compared the stem-like properties of subpopulations with different combination of the expression status of NADH and CD133/CD15, including NADH^high^/CD133^+^, NADH^high^/CD133^−^, NADH^low^/CD133^+^, NADH^low^/CD133^−^, NADH^high^/CD15^+^, NADH^high^/CD15^−^, NADH^low^/CD15^+^, and NADH^low^/CD15^−^. NADH^high^/CD133^+^ subpopulation showed the highest expression of stem-related genes Nanog, Oct4, Oligo2, and Sox2, whereas NADH^low^/CD133^−^ subpopulation had the lowest expression of those genes in GBM1 and LN229 cells (Fig. 4a). Compared to NADH^high^/CD133^+^ and NADH^low^/CD133^−^ subpopulations, both NADH^high^/CD133^−^ and NADH^low^/CD133^+^ subpopulations exhibited medium expression levels of those genes (Fig. 4a). Similar results were observed in the subpopulations which combined NADH and CD15 markers (Additional file 1: Figure S5A). Correspondingly, NADH^high^/CD133^+^ subpopulation had the highest ability of tumorsphere formation, NADH^low^/CD133^−^ subpopulation had the lowest ability of tumorsphere formation, and NADH^high^/CD133^−^ and NADH^low^/CD133^+^ subpopulations exhibited the medium ability of tumorsphere formation in GBM1 and LN229 cells (Fig. 4b). Besides, compared to the diameter, the average diameter of tumorspheres derived from NADH^high^/CD133^+^, NADH^high^/CD133^−^, and NADH^low^/CD133^+^ was markedly larger than that of NADH^low^/CD133^−^-derived tumorspheres in GBM1214 and LN229 (Fig. 4c). The similar results were observed in the subpopulations which combined NADH and CD15 markers (Additional file 1: Figure S5B and C). Therefore, these results suggest that NADH^high^ subpopulation exhibits similar stemness traits with CD133^+^ and CD15^+^ subpopulations in vitro.

### The invasion ability and temozolomide resistance of NADH^high^ subpopulation are comparable with CD133^+^ and CD15^+^ subpopulations in glioma cells

**Fig. 5:**
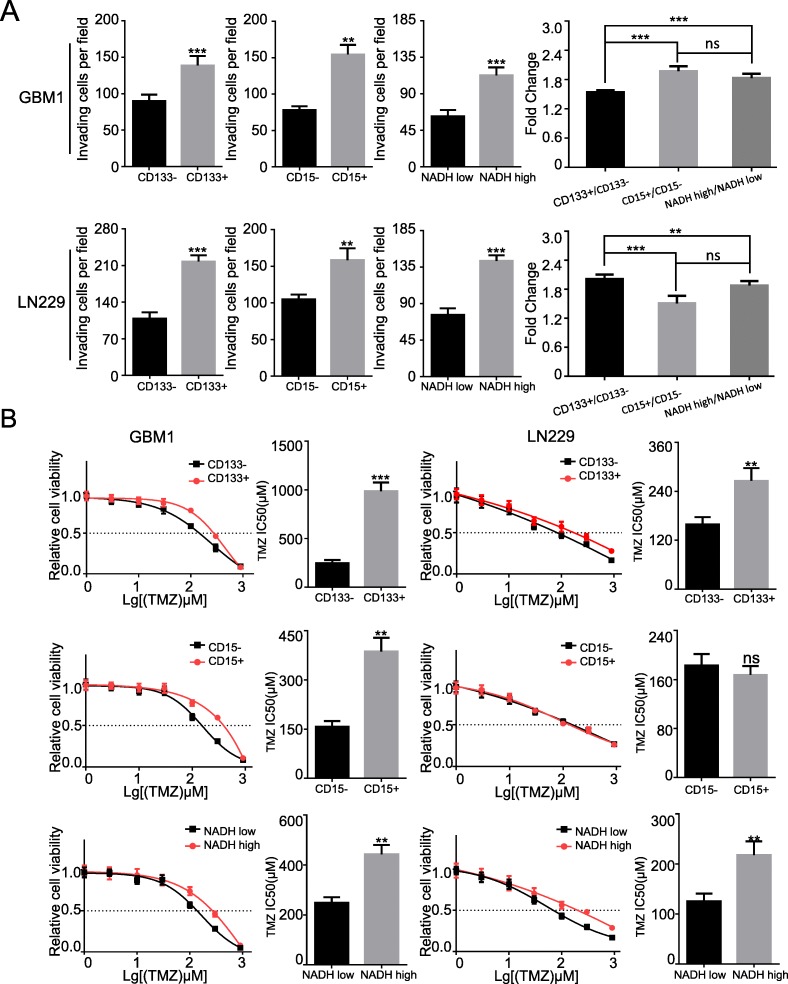
The invasion ability and TMZ resistance of NADH^high^ glioma cells are similar to the ability of CD133^+^ and CD15^+^ subpopulation in glioma cells. **a** The quantitative histograms of invasion showed that invasion ability was significantly increased in NADH^high^, CD133^+^, and CD15^+^ LN229 and GBM1 cells. **b** Effect of TMZ resistance in CD133, CD15, and NADH. IC50 of TMZ in CD133^+^ and NADH^high^ was higher than in CD133^−^, and NADH^low^ of GBM1 and LN229. The similar result was in CD15 of GBM1, but IC50 of TMZ in CD15 of LN229 was not different. All data are presented as the means ± SD. **p < 0.01, ***p < 0.001 (*n* = 3 independent experiments)

Previous studies have demonstrated that GSCs are implicated in tumor invasiveness and chemotherapeutic resistance [33, 34]. Compared with NADH^low^ subpopulation, NADH^high^ subpopulation had higher invasive ability in LN229 and GBM1 cells (*p* < 0.01 for both) (Fig. 5a). The invasive abilities between NADH^high^, CD133^+^, and CD15^+^ subpopulations were comparable (*p* < 0.01) (Fig. 5a and Additional file 1: Figure S8). Moreover, NADH^high^ cells were less sensitive to TMZ than NADH^low^ cells (Fig. 5b). CD133^+^ and CD15^+^ cells were more resistant to TMZ than CD133^−^ and CD15^−^ cells (Fig. 5b), which were consistent with the previous reports [33, 35]. These results suggest that NADH^high^ subpopulation has similar malignant behaviors of invasion and chemotherapeutic resistance with CD133^+^ and CD15^+^ subpopulations.

### The intensity of NADH autofluorescence can be used as a biomarker to sort other CSCs

**Fig. 6:**
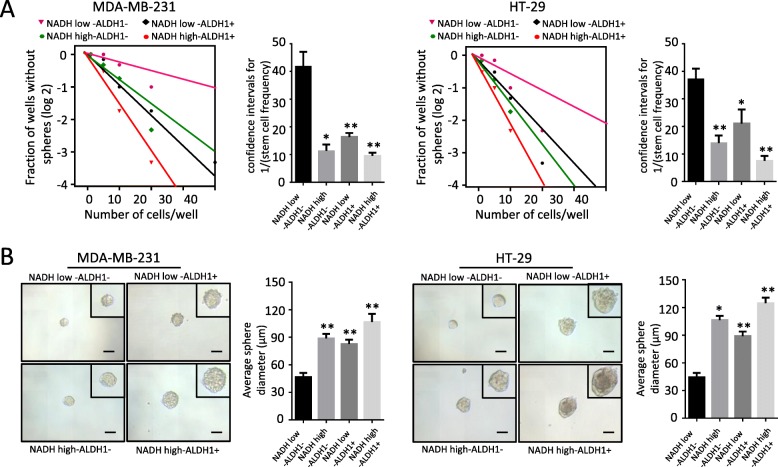
The intensity of NADH autofluorescence as a biomarker can be used to sort breast cancer stem cells and colon cancer stem cells. **a** Limiting dilution showed that compared to NADH^low^/ALDH^−^ cells, NADH^high^/ALDH^+^, NADH^high^/ALDH^−^, and NADH^low^/ALDH^+^ have higher sphere formation in MDA-MB-231 and HT-29. **b**, **c** The average diameter of the tumorspheres in NADH^high^/ALDH^hig^h, NADH^high^/ALDH^low^, and NADH^low^/ALDH^high^ subpopulations was larger than that in NADH^low^/CD133^−^ subpopulations in MDA-MB-231 and HT-29 cells. All data are presented as the means ± SD. **p < 0.01, *p < 0.05 (*n* = 3 independent experiments)

In order to assess whether the intensity of NADH autofluorescence was suitable for isolating the CSCs in other tumors, we sorted NADH^high^ and NADH^low^ subpopulations from breast cancer cell line MDA-MB-231 and colorectal cancer cell line HT-29. With limiting dilution, we evaluated the self-renewal capability between NADH^high^/ALDH^+^, NADH^high^/ALDH^−^, NADH^low^/ALDH^+^, and NADH^low^/ALDH^−^ cells. Compared to NADH^low^/ALDH^−^ subpopulation, NADH^high^/ALDH^+^, NADH^high^/ALDH^−^, and NADH^low^/ALDH^+^ exhibited higher ability of tumorsphere formation both in MDA-MB-231 and HT-29 (Fig. 6a). Besides, the average diameter of the tumorspheres derived from NADH^high^/ALDH^+^, NADH^high^/ALDH^−^, and NADH^low^/ALDH^+^ was larger than that from NADH^low^/ALDH^−^ both in MDA-MB-231 and HT-29 (Fig. 6b). Thus, the intensity of NADH autofluorescence could be used as a biomarker to isolate CSCs from breast cancer and colorectal cancer, implying that the intensity of NADH autofluorescence might be an extensive biomarker for CSCs.

## Discussion

Many endogenous ingredients of cells and tissues, such as some amino acids, collagen, elastin, NAD(P) H, flavin adenine dinucleotide (FAD), vitamins, lipids, and porphyrins, possess natural autofluorescence [36, 37]. Because these endogenous autofluorescence ingredients are the metabolites of cells or tissues, their autofluorescence intensity may directly reflect the physiological and/or pathological status of cells and tissues. So far, only the autofluorescence of NAD(P) H and FAD has been widely studied, mainly to be used in monitoring alteration of metabolic profiles and cellular oxidation-reduction status [38–40]. Moreover, the autofluorescence of NAD(P) H and FAD has been studied in normal stem cells and CSCs. Quinn et al. reported that the quantitative metabolic imaging using the endogenous fluorescence of NADH and FAD could monitor human mesenchymal stem cell differentiation into adipogenic and osteoblastic lineages [41]. Fluorescence of free and protein-bound NADH could discriminate different differentiation stages of neuronal progenitor stem cells [42]. Buschke et al. used multiphoton flow cytometry to non-invasively characterize and purify populations of intact stem cell aggregates based on NADH intensity and assessed the differentiation capacity of sorted populations [43]. Bonuccelli et al. demonstrated that NAD(P) H autofluorescence was a new metabolic biomarker for CSCs in MCF-7 breast cancer cell line and sorted high NAD(P) H autofluorescence intensity cells exhibited CSC phenotype [26]. Miranda-Lorenzo et al. used FAD autofluorescence as a novel tool to isolate and characterize epithelial CSCs, but it had obvious limitations, such as exogenous riboflavin needed to be added to enhance the sensitivity, and the experimental results varied with the concentrations of riboflavin, incubation times, and cell concentrations [44]. Therefore, in comparison with FAD, NADH autofluorescence is a more reliable and promising biomarker to be used to sort CSCs without exogenous substances to be added. In the present study, we sorted NADH^high^ subpopulation from glioma cells and further demonstrated that this subpopulation possessed the properties of CSCs, featured with significant increase of stemness-related gene expression, tumorsphere formation, invasiveness, resistance to TMZ in vitro, and tumorigenicity in vivo.

Herein, we used a wavelength of 355 nm or 375 nm for the autofluorescence of NADH. However, under our experimental conditions, the sorted NADH^high^ subpopulation actually also contained NADPH^high^ cells because NADH and NADPH are spectrally identical. Nevertheless, despite the two co-enzymes exert different functions with NAD/NADH as a key determinant of cellular energy metabolism and NADP/NADPH as a central role in biosynthetic pathways and antioxidant defense, both of them may be important for stemness maintenance of CSCs. Several other studies have suggested that the concentration of NADH is higher (up to 5 times) than the NADPH in mammalian and the quantum yield of NADH is 1.25 to 2.5 times higher than that of NADPH [45]. Since NADH is the main source of the autofluorescence, we used NADH^high^ but not NAD(P) H^high^ subpopulation as GSCs.

CD133 and CD15 have been regarded as reliable maker for enriching GSCs. In our studies, we compared the relationship of CD133^+^, CD15^+^, and NADH^high^ subpopulations and found that CD133/CD15 defines distinct cell subpopulations and both CD133^+^ and CD15^+^ cells were only partially overlapped with NADH^high^ subpopulation in glioma cells. Thus, NADH^high^ may define a subset of GSCs independent of CD133^+^ and CD15^+^ subsets. As for the relationship between CD133^+^ and CD15^+^ cells, Son et al. reported that most CD133^+^ tumor cells freshly isolated from glioma specimens were CD15^+^ [6], but a less overlap between CD133^+^ and CD15^+^ subsets was observed in GBM1 and LN229 cells (Additional file 1: Figures S6 and S7).

As a basic metabolite, NADH is ubiquitously distributed in cells. Therefore, NADH autofluorescence could be a biomarker not only for GSCs, but also for other CSCs. Indeed, we found that NADH^high^/ALDH^+^, NADH^high^/ALDH^−^, and NADH^low^/ALDH^+^ subpopulations had higher self-renewal ability than NADH^low^/ALDH^−^ subpopulation in breast cancer and colon cancer cells, implying that the autofluorescence of NADH might serve as a biomarker for CSCs of these cancers.

## Conclusion

Our findings demonstrate that intracellular autofluorescence of NADH is a non-labeling, sensitive maker for isolating GSCs, even for other CSCs.

## Supplementary information

**Additional file 1: Table S1.** The clinical features of the glioma specimens used in this study, **Table S2.** Primers used for qRT-PCR analyses in this study. **Table S3.** Tumor formation rates of xenograft implanted with different cell number of NADH^high^ and NADH^low^ LN229 cells. **Figure S1.** Intensity of NADH autofluorescence in different WHO grade glioma tissues detected by FACS. **Figure S2.** Representative images of flow cytometry analysis for proportion of CD133+ cells in NADH^high^ and NADH^low^ subpopulations in glioma cells. **Figure S3.** Representative images of flow cytometry analysis for proportion of CD15+ cells in NADH^high^ and NADH^low^ subpopulations in glioma cells. **Figure S4.** The representative flow cytometry images of the percentage of NADH^high^ cells in CD133+/CD15+ populations. **Figure S5.** Both NADH^high^ and CD15+ giloma cells possess the properties of CSCs, but are partially overlapped. **Figure S6.** The representative flow cytometry images of the relationship between CD133^+^, CD15^+^ and NADH^high^ populations. **Figure S7.** The representative flow cytometry images of the relationship between CD133^+^, CD15^+^ and NADH^high^ populations. **Figure S8.** The representative images of invasion assay for NADH^high^ and NADH^low^, CD133^+/-^ and CD15^+/-^ subpopulations in GBM1 and LN229 cell lines. Compared to CD133^-^, CD15^-^ NADH^low^ subsets, CD133^+^, CD15^+^ and NADH^high^ cells exhibited stronger invasive ability in GBM1 and LN229 cell lines.

## Data Availability

For data requests, please contact the authors.

## Abbreviations

ALDH: Aldehyde dehydrogenase
bFGF: Basic fibroblast growth factor
CSCs: Cancer stem cells
EGF: Epidermal growth factor
FACS: Fluorescence-activated cell sorting
FAD: Flavin adenine dinucleotide
FLIM: Fluorescence lifetime microscopy
GSCs: Glioma stem cells
NADH: Nicotinamide adenine dinucleotide
TMZ: Temozolomide
WHO: World Health Organization
