# Supplementary material for: Autofluorescence of NADH is a new biomarker for sorting and characterizing cancer stem cells in human glioma
Source: Stem Cell Res Ther. 2019 Nov 20;10:330. doi: 10.1186/s13287-019-1467-7 (PMC6865050; doi:10.1186/s13287-019-1467-7)
Supplement: Supplementary file 1 — Additional file 1: Table S1. The clinical features of the glioma specimens used in this study, Table S2. Primers used for qRT-PCR analyses in this study. Table S3. Tumor formation rates of xenograft implanted with different cell number of NADHhigh and NADHlow LN229 cells. Figure S1. Intensity of NADH autofluorescence in different WHO grade glioma tissues detected by FACS. Figure S2. Representative images of flow cytometry analysis for proportion of CD133+ cells in NADHhigh and NADHlow subpopulations in glioma cells. Figure S3. Representative images of flow cytometry analysis for proportion of CD15+ cells in NADHhigh and NADHlow subpopulations in glioma cells. Figure S4. The representative flow cytometry images of the percentage of NADHhigh cells in CD133+/CD15+ populations. Figure S5. Both NADHhigh and CD15+ giloma cells possess the properties of CSCs, but are partially overlapped. Figure S6. The representative flow cytometry images of the relationship between CD133+, CD15+ and NADHhigh populations. Figure S7. The representative flow cytometry images of the relationship between CD133+, CD15+ and NADHhigh populations. Figure S8. The representative images of invasion assay for NADHhigh and NADHlow, CD133+/- and CD15+/- subpopulations in GBM1 and LN229 cell lines. Compared to CD133-, CD15- NADHlow subsets, CD133+, CD15+ and NADHhigh cells exhibited stronger invasive ability in GBM1 and LN229 cell lines. [file 13287_2019_1467_MOESM1_ESM.docx]

**Supplementary Tables**

**Table S1.** **The clinical features of the glioma specimens used in this study.**

| **Feature** | **Number (Percentage)** |
| --- | --- |
| Gender |  |
| Male | 8(57.1%) |
| Female | 6(42.9%) |
| Age (year, median ± SD) | 42.8±18.4 |
| Grade |  |
| Ⅱ | 4(29.6%) |
| Ⅲ | 3(21.4%) |
| Ⅳ | 7(50%) |
|  |  |
| Predominant side of tumor location |  |
| Left | 7(50%) |
| Right | 6(42.9%) |
| Middle | 1(7.1%) |
| Predominant lobe of tumor location |  |
| Frontal | 8(57.1%) |
| Temporal | 3(21.4%) |
| Parietal | 2(14.4%) |
| others | 1(7.1%) |

Abbreviation: SD, Stardard deviation.

**Table S2.** **Primers used for qRT-PCR analyses in this study.**

| Gene | Forward primer (5' to 3') | Reverse primer (5' to 3') |
| --- | --- | --- |
| *CD133* | AGTCGGAAACTGGCAGATAGC | GGTAGTGTTGTACTGGGCCAAT |
| *SOX2* | GCCGAGTGGAAACTTTTGTCG | GGCAGCGTGTACTTATCCTTCT |
| *NANOG* | TTTGTGGGCCTGAAGAAAACT | AGGGCTGTCCTGAATAAGCAG |
| *OCT4* | CTGGGTTGATCCTCGGACCT | CCATCGGAGTTGCTCTCCA |
| [*OLIG2*](https://www.ncbi.nlm.nih.gov/gene/203068) | CCAGAGCCCGATGACCTTTTT | CACTGCCTCCTAGCTTGTCC |
| [*TUBB*](https://www.ncbi.nlm.nih.gov/gene/203068) | TGGACTCTGTTCGCTCAGGT | TGCCTCCTTCCGTACCACAT |

**Table S3.** Tumor formation rates of xenograft implanted with different cell number of NADH^high^ and NADH^low^ LN229 cells.

| Cell number | NADH^high/low^ group | Tumor incidence |
| --- | --- | --- |
| 4 × 10^3^ | NADH^high^ | 4/5(80%) |
|  | NADH^low^ | 1/5(20%) |
| 4×10^4^ | NADH^high^ | 5/5(100%) |
|  | NADH^low^ | 3/5(60%) |
| 4×10^5^ | NADH^high^ | 5/5(100%) |
|  | NADH^low^ | 5/5(100%) |

**Supplementary Figures**

**Figure S1: Intensity of NADH autofluorescence in different WHO grade glioma tissues detected by FACS.**

**
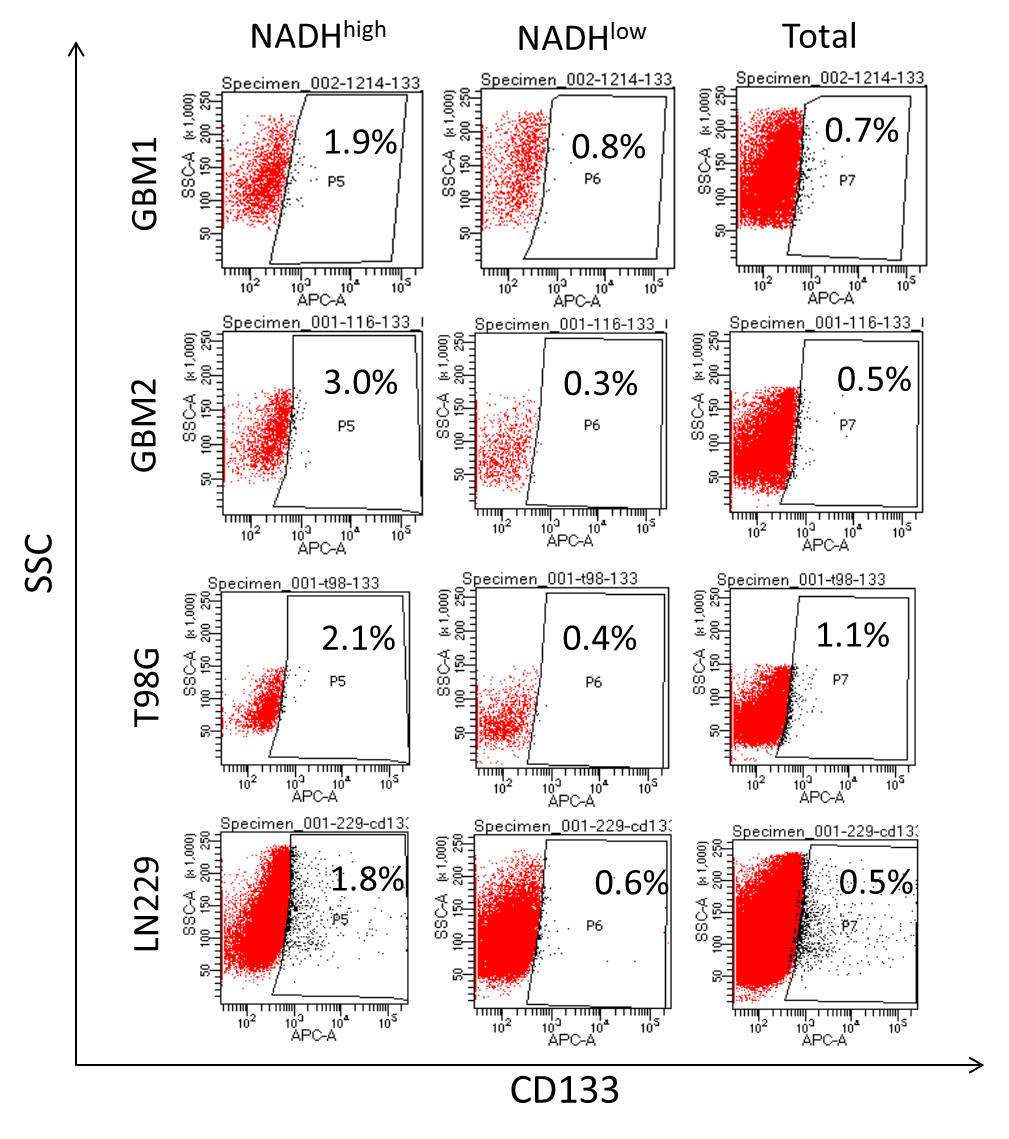
**

**Figure S2: Representative images of flow cytometry analysis for proportion of CD133+ cells in NADH^high^ and NADH^low^ subpopulations in glioma cells.** The percentage of CD133+ cells was higher in NADH^high^ population than in NADH^low^ population and total population.


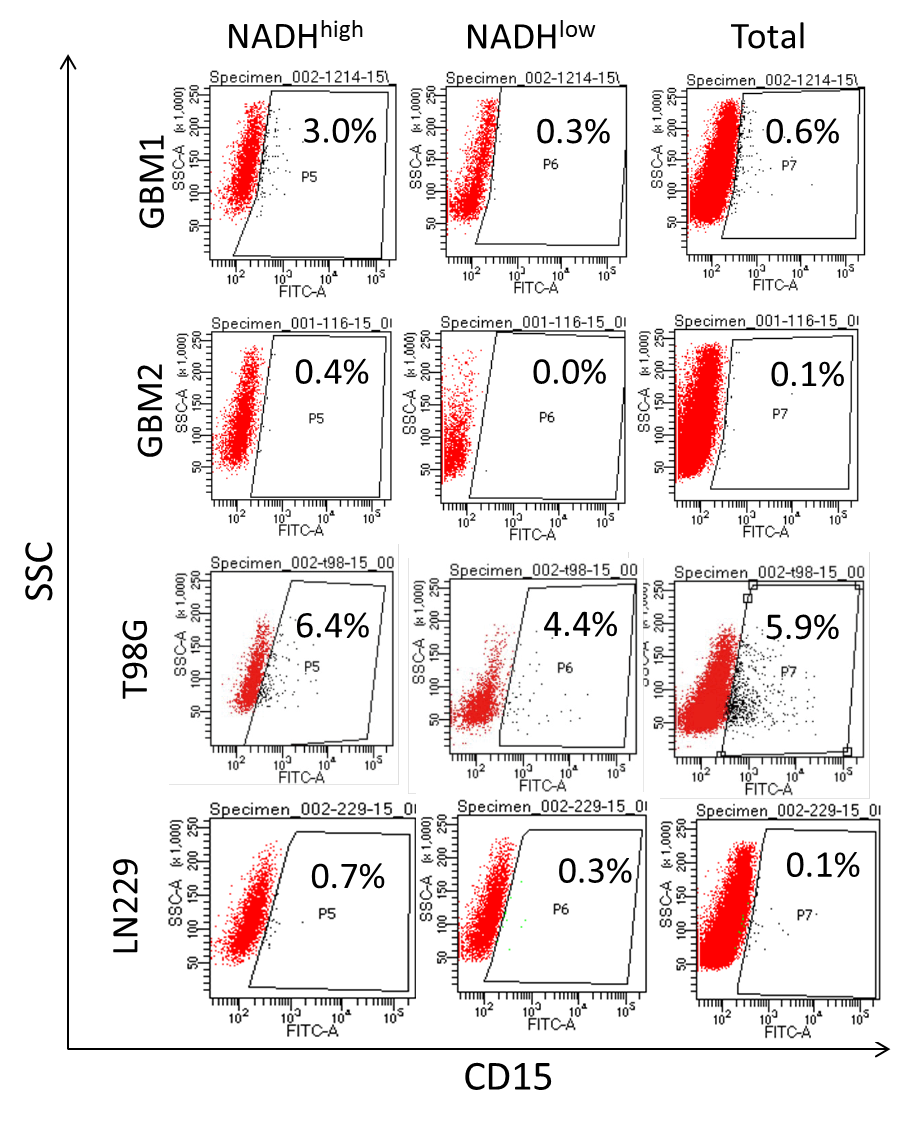


**Figure S3: Representative images of flow cytometry analysis for proportion of CD15+ cells in NADH^high^ and NADH^low^ subpopulations in glioma cells.** The percentage of CD15+ cells was higher in NADH^high^ population than in NADH^low^ population and total population.


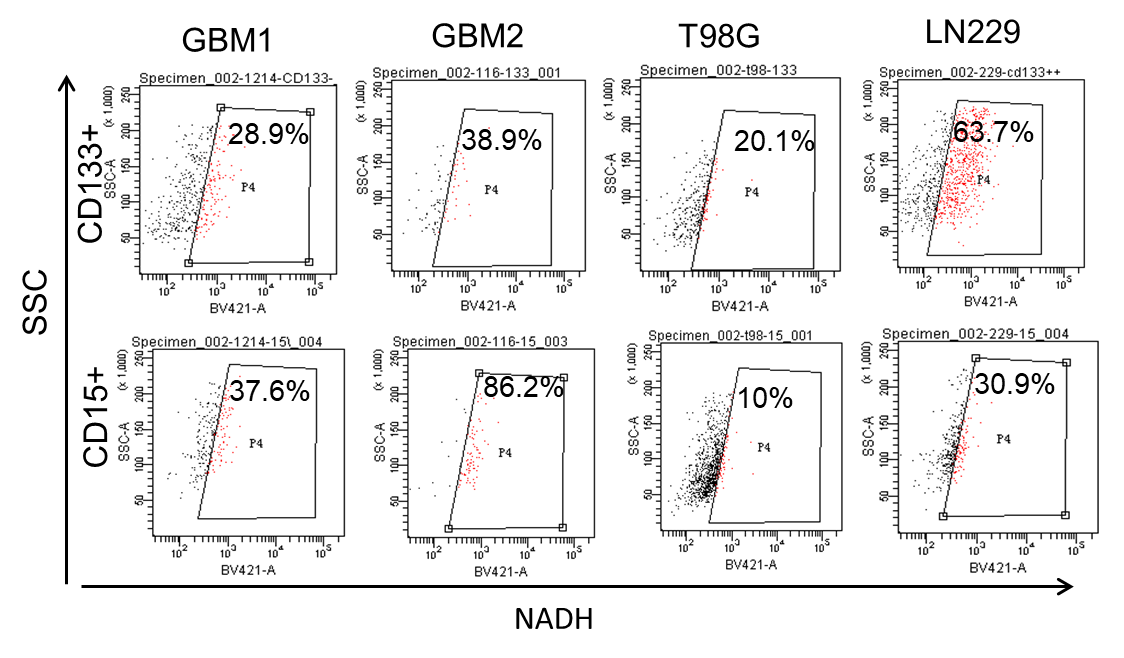


**Figure S4: The representative flow cytometry images of the percentage of NADH^high^ cells in CD133+/CD15+ populations.**


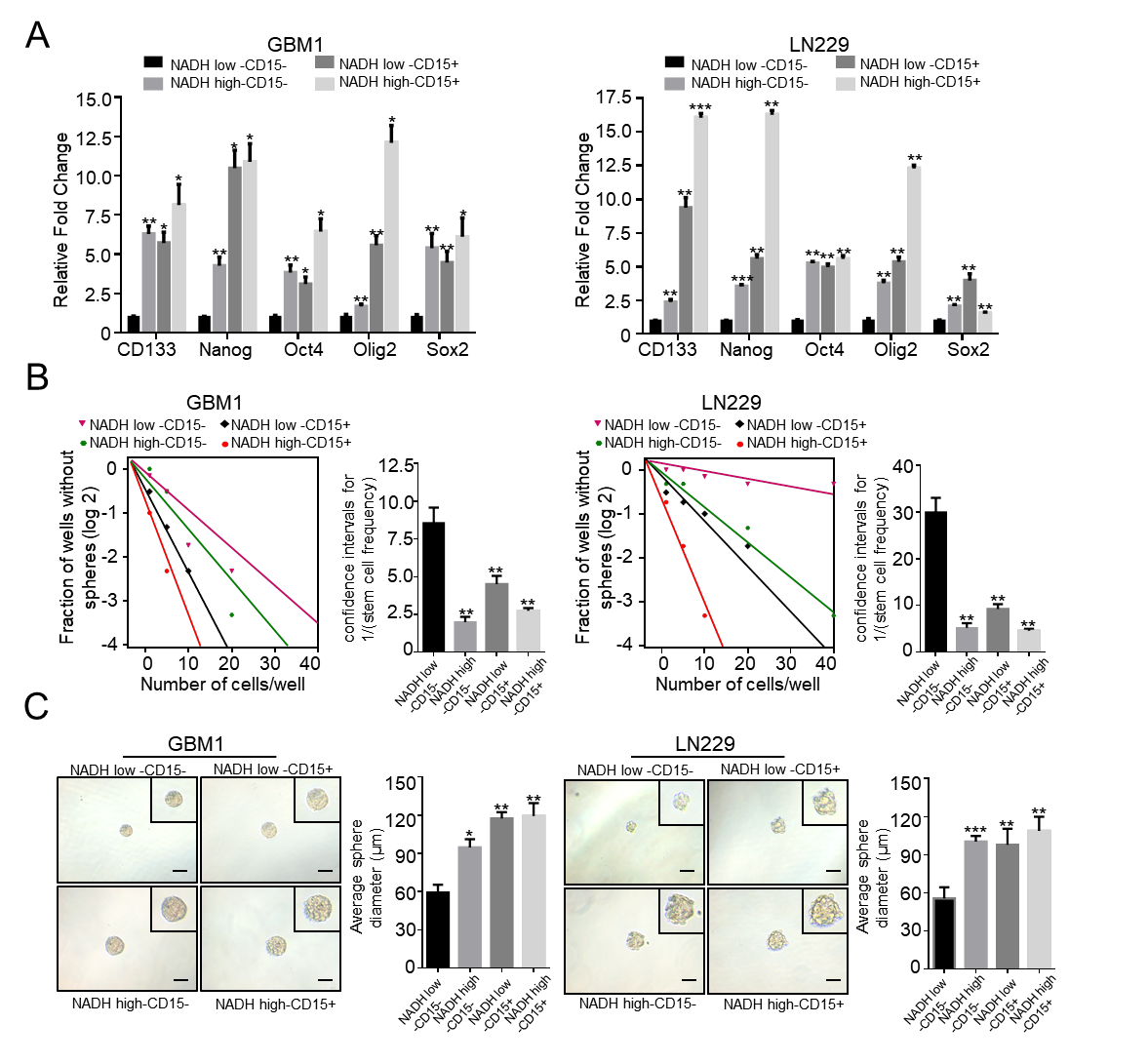


**Figure S5: Both NADH^high^ and CD15+ giloma cells possess the properties of CSCs, but are partially overlapped.** (A) ) qRT-PCR assays showed that the expression levels of stemness-related transcription factor genes Nanog, Oct4, Oligo2 and Sox2 were the highest in NADH^high^/CD15^+^ subpopulations, medium in NADH^high^/CD15^-^ and NADH^low^/CD15^+^ subpopulation, and the lowest in NADH^low^/CD15^-^ subpopulation in GBM1 and LN229 cells. (B) Limiting dilution showed that the ability of sphere formation was the highest in NADH^high^/CD15+ subpopulations, medium in NADH^high^/CD15^-^ and NADH^low^/CD15^+^ subpopulations, and the lowest in NADH^low^/CD15^-^ subpopulation in GBM1 and LN229 cells. (C) The spheres derived from NADH^high^/CD15^+^ cells had the maximum diameter and the spheres derived from NADH^low^/CD15^-^ cells had the minimum diameter, while the spheres derived from NADH^high^/CD15^-^ and NADH^low^/CD15^+^ subpopulations had a medium diameter. . All data are presented as the means ± SD. **P* < 0.05, ***P* < 0.01,****P* < 0.001. (n = 3 independent experiments)


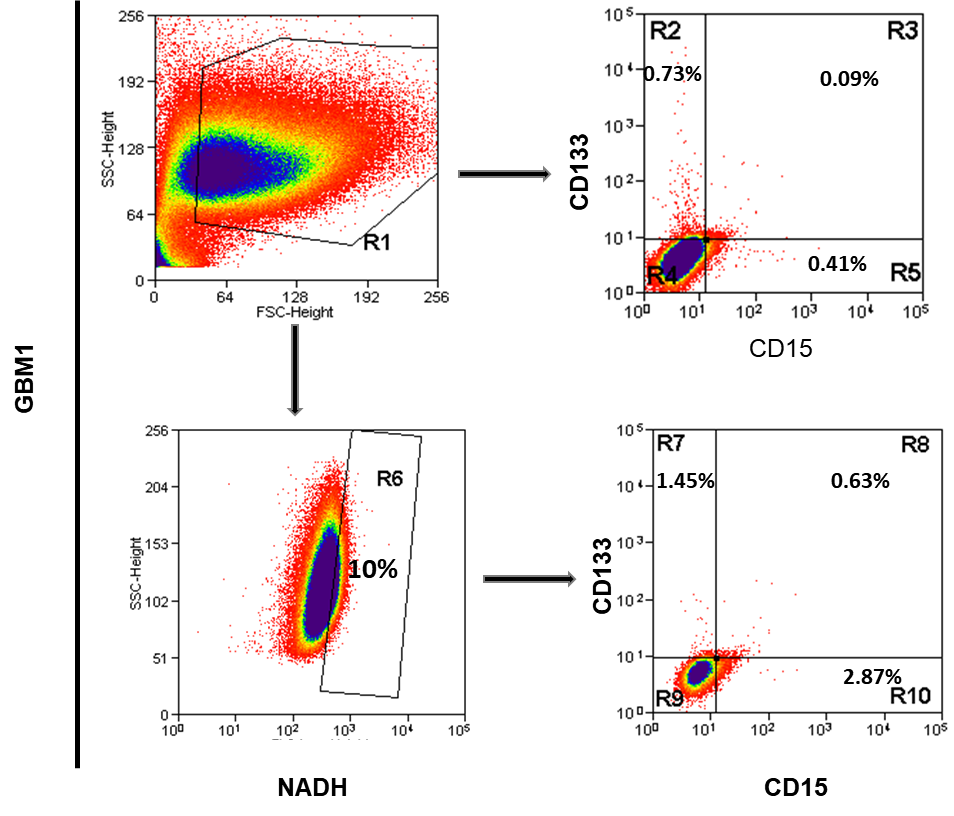


**Figure S6: The representative flow cytometry images of the relationship between CD133^+^, CD15^+^ and NADH^high^ populations.** CD133^+^ and CD15^+^ subsets were partially enriched in NADH^high^ population, but they were independent of each other in total population and NADH^high^ population in GBM1 cells.


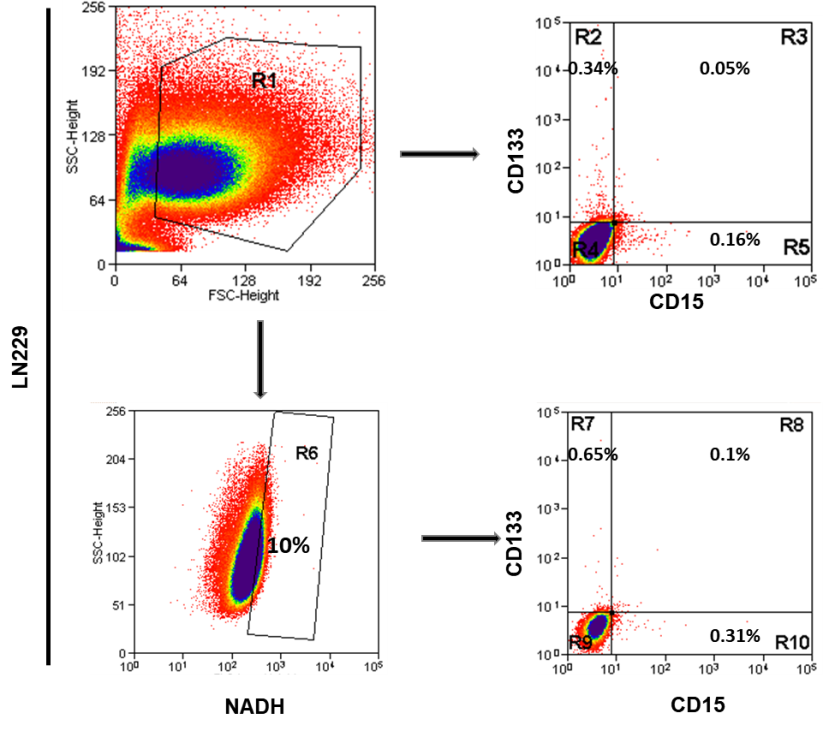


**Figure S7: The representative flow cytometry images of the relationship between CD133^+^, CD15^+^ and NADH^high^ populations.** CD133^+^ and CD15^+^ subsets were partially enriched in NADH^high^ population, but they were independent of each other in total population and NADH^high^ population in LN229 cells.


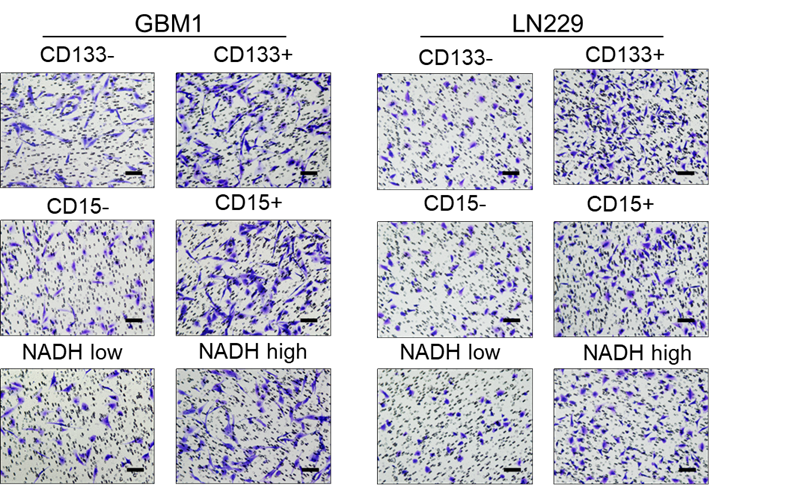


**Figure S8: The representative images of invasion assay for NADH^high^ and NADH^low^, CD133^+/-^ and CD15^+/-^ subpopulations in GBM1 and LN229 cell lines.** Compared to CD133^-^, CD15^-^ NADH^low^ subsets, CD133^+^, CD15^+^ and NADH^high^ cells exhibited stronger invasive ability in GBM1 and LN229 cell lines.
